# Noninvasive blood glucose monitoring using a dual band microwave sensor with machine learning

**DOI:** 10.1038/s41598-025-94367-6

**Published:** 2025-05-09

**Authors:** Mariam Farouk, Anwer S. Abd El-Hameed, Angie R. Eldamak, Dalia N. Elsheakh

**Affiliations:** 1 Electrical Department, Faculty of Engineering and Technology, Badr University in Cairo, Badr, 11829 Egypt; 2 Microstrip Department, Electronics Research Institute (ERI), El Nozha, Giza, 4473221 Egypt; 3 Electronics and Communications Engineering Department, Faculty of Engineering, Ain Shams University, Cairo, 11517 Egypt

**Keywords:** Biotechnology, Health care, Engineering

## Abstract

The potential for continuous non-invasive blood glucose monitoring has attracted a lot of interest in the field of medical diagnostics. This paper provides a new shape of a dual-band bandpass filter (DBBPF) acting as a microwave transmission line sensor for continuous non-invasive blood glucose monitoring operating at 2.45 and 5.2 GHz. The proposed system uses the interaction between biological tissues and microwave signals to correctly assess blood glucose levels. The proposed dual-band bandpass filter (DBBPF), comprises three split ring resonator (SRR) cells with different dimensions. It is designed to operate as a sensor with improved sensitivity, compact dimensions, and a high-quality factor. It also ensures a reasonable bandwidth for lower and higher bands of 8.6 and 2%, respectively in the industrial, scientific, medical band, and the wireless local area network (ISM and WLAN) Bands. A dual-band filter enhances measurement sensitivity and specificity by targeting specific frequency ranges where glucose exhibits distinctive dielectric responses, thereby providing redundant data points for accurate glucose level determination. Glucose concentrations can be evaluated by measuring the changes in the dielectric properties of blood by sending microwave waves through the body and assessing the collected S-parameter signals. The measurement parameters encompass the reflection, phase, magnitude, as well as transmission parameters. This yields multiple evaluations of the glucose-induced alterations. Simulations are validated through laboratory measurements incorporating a phantom finger model for capturing realistic outcomes. Machine learning models are employed to analyze the sensor data, improving the accuracy of diabetes detection. Simulations are validated through laboratory measurements incorporating a phantom finger model for capturing realistic outcomes. A Cole-Cole model, implemented using MATLAB, is utilized for the phantom finger model. The main results reveal the success of the proposed transmission-based microwave glucose sensing, with a remarkable sensitivity of 1 ~ 1.5 dB for glucose level change up to 200 mg/dL.

## Introduction

Diabetes is a progressive, lifelong illness that, regrettably, affects millions of people worldwide^1^. It is one of the most serious long-term illnesses, characterized by abnormally elevated blood glucose levels that are frequently controlled by the pancreatic hormone insulin^2^. Type 1 diabetes in people is caused by insufficient insulin production by the pancreas, while type 2 diabetes develops when insulin resistance occurs^3^.

Accordingly, a person’s eating schedule and overall health determine how much glucose is in their blood. When fasting (that is, not eating for at least eight hours) in healthy persons, blood glucose levels range from 70 to 99 mg/dL (3.9 to 5.4 mmols/L) and these values range from 90 to 110 mg/dL (or 5 to 6.1 mmols/L) two hours after eating^4^. Long-term high blood glucose levels can cause major consequences like heart palpitations, stroke, blindness, visual issues, and kidney disease^1^. Conversely, low blood glucose levels can raise the risk of conditions like kidney disease and eye diseases. Low blood glucose, on the other hand, raises the risk of several illnesses, including renal disease, nerve damage, and eye problems. In extreme circumstances, low glucose delivery may cause nerve cells to continue to die^5^.

**Fig. 1:**
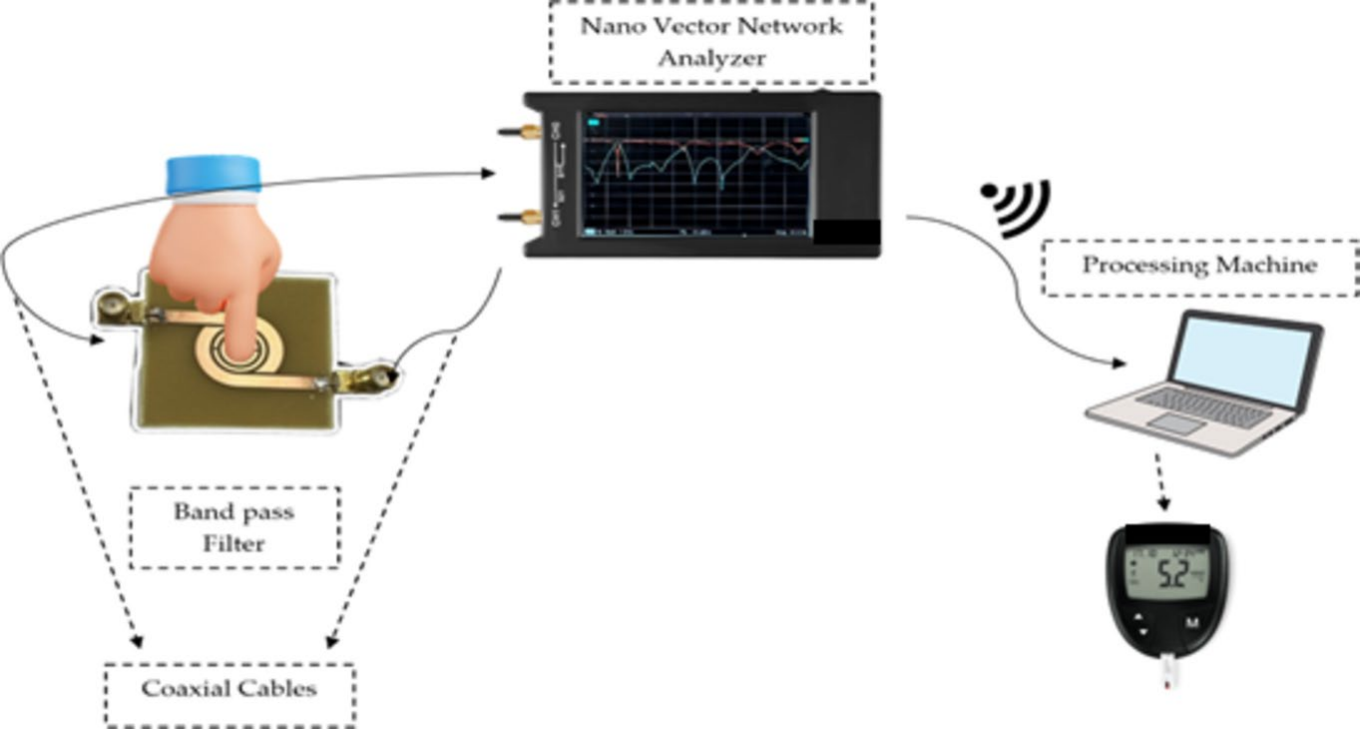
The system is composed of SRR targeting finger tissues.

In the proposed sensor as shown in (Fig. 1), a dual-band bandpass filter (DBBPF) as a sensor is the host and modified SRRs are the detecting region. The input/output coupling method^6,7^ for (DBBPF) functioning is designed to respond near the ISM/WLAN band. The sensor’s architecture makes it more resistant to noise and environmental influences. The suggested sensor’s operating band, known as the ISM band, is a license-free band used in buildings, home automation, or hospitals^8^.

The most common tools for monitoring diabetes are currently glucometers, which are self-monitoring blood glucose devices based on the continuous glucose monitoring (CGM) technique. As a result, self-monitoring blood glucose monitors use a lancet to measure the glucose concentration in a drop of blood on a test strip via finger prick. Through the use of an implanted sensor beneath the skin, usually in the thigh, abdomen, or upper arm, the CGM also keeps track of blood glucose levels^8,9^. The current blood glucose monitoring instruments are invasive, uncomfortable, and costly, which has led to the development of non-invasive measurement approaches in recent years^10^. Thus, individuals with diabetes will have a significant improvement in their quality of life, pain-free diabetic monitoring, and reduced financial issues resolved using non-invasive blood glucose monitoring techniques. These techniques include optical, transdermal, and microwave^11^. Providing non-invasive devices to measure glucose concentrations helps to lessen the environmental impact^12^ and can directly interact with glucose concentrations without the need for replaceable parts^9^. Microwave radiation will reach deeper into tissues than other conventional methods including scattering, photoacoustic, Raman, and near-infrared spectroscopy^13,14^. Moreover, Microwave devices are perfect for non-invasive measurements because of their small size, ease of fabrication, low cost, and strong penetration depth^9,13^.

By tracking changes in conductivity and dielectric constant, microwave sensors are used to assess changes in blood glucose levels. Studies have demonstrated that glucose levels may be measured using microwave sensors operating at various frequencies^15,16^. Split-ring resonators (SRRs)^17–20^, complementary electric-LC resonators (CELCRs)^21^, patch antennas^22^, dielectric resonator antennas^23^, patch resonators^24^, open-loop microstrip resonators^25^, spiral microstrip resonators^26^, and complementary SRRs (CSRRs)^27–30^ There are a few types of microwave sensors.

Many sensing metrics, including resonant frequency (f) shifts, transmission/reflection coefficient magnitude and phase, (S21/S11), and quality factor (Q factor)^15,31^, are frequently used to evaluate the sensitivity of microwave sensors. In some experiments, these characteristics can be obtained from a single measurement^31^. While some studies have examined sensitivity by changing the phase in^31^. Several studies have assessed sensitivity to changes in resonant frequency and scattering S-parameter amplitude. With this method, measurements may be made with greater accuracy and microwave sensor applications can function better. Several techniques, such as interdigital capacitance^32–34^, closed-loop resonators, and microstrip resonators, have been employed to identify glucose in aqueous solutions^35,36^. Additionally, researchers have improved microwave sensor performance by using 3D printing techniques to identify glucose^37^. Based on unloaded Q factor sensing with a single SSR, a novel biocompatible glucose sensor with a distinctive design has been created^38^. Based on input impedance, this microstrip line-based sensor indicates blood glucose levels and enhances glucose detection in aqueous solutions^37^. RF sensors are gaining increasing attention for monitoring biochemical components due to their non-invasive nature, rapid response times, and potential for continuous monitoring. Recent advancements have demonstrated their effectiveness in various applications, such as glucose monitoring, pH level detection, and hydration assessment. For instance, Ref^38–40^. have shown promising developments in RF sensor technology for biochemical sensing, with improved sensitivity, miniaturization, and integration capabilities. These advancements underscore the growing importance of RF sensors in healthcare and biomedical applications, further supporting the relevance of our research on non-invasive glucose monitoring using RF-based techniques.

Large amounts of glucose data gathered over time can be analyzed by machine learning technology. machine learning models that process this data can find connections, trends, and patterns that people would find difficult to see. Insights on a person’s glucose trends can be gained from this study, which can improve diabetes treatment plans^41^. Artificial intelligence-based systems can forecast future glucose levels based on past glucose data. With the use of this predictive power, people may take proactive measures to control their blood sugar levels by changing their food, how much insulin they take, or how much exercise they get^42^. Moreover, by analyzing glucose data and producing suggestions for therapy modifications, machine learning models could assist medical personnel in making decisions. This can help medical professionals make well-informed choices on when and how much to prescribe medications, among other treatments^43^. Furthermore, artificial intelligence makes it possible to check glucose levels remotely. machine learning-enabled non-invasive glucose monitoring devices can provide real-time glucose data to medical specialists, enabling remote monitoring and prompt actions. People who reside in distant places or have restricted access to medical services would especially benefit from this. Thus, machine learning may be used with other technologies to offer a complete diabetes control solution, including wearables and mobile apps. For instance, wearable technology, such as activity trackers or smartwatches, can supply data to machine learning models that can be used to provide customized suggestions for controlling blood sugar levels.

This paper presents a non-invasive sensor based on a microwave dual-band bandpass filter transmission line that can measure glucose concentrations in vitro. The relative permittivity and loss tangent of solutions containing different blood glucose levels are assessed in the first stage to characterize the glucose variations to the microwave frequencies. In the second step, the S-parameters of the resonant frequency, the magnitude and phase of the reflection, and the transmission coefficient are chosen to determine the sensitivity of the sensors. These parameters are extracted from both the simulations and measurement results of the proposed sensor, loaded with different glucose concentration levels. This paper uses a novel shape of a dual bandpass filter integrated with a nano vector network analyzer to design the proposed system of monitoring glucose in humans by using the non-invasive method as shown in (Fig. 1). This paper presents an improved approach for using microwave technology, with an SRR design for precise detection of glucose solutions at different concentrations. The design of a dual bandpass filter based on the SRR method enables the detection of glucose concentrations in several frequency bands, potentially improving the accuracy and reliability of the readings. It also improves the sensor’s quality factor (Q-factor) performance. The reported sensitivity values (e.g., 0.00067 degrees/(mg/dL) and 2.026 MHz/(mg/dL)) demonstrate that the proposed sensor can detect fine variations in glucose levels, including states of hypoglycemia, normoglycemia, and hyperglycemia. This level of sensitivity is particularly crucial for effective diabetes management. The proposed methodology connects glucose concentration with S-parameter magnitude, phase, and shift in resonant frequency to determine the biosensing response and add depth to the interpretation of microwave sensing data. Moreover, adding machine learning models such as Random Forest and CatBoost classifiers to the conventional sensing procedure enhances its forecast precision and adjusts to different glucose concentrations. The proposed sensor acquires a low-profile, low-cost design that makes the technology accessible and practical for widespread use.

The organization of this paper is as follows: Sect. 1 shows the introduction, explains the importance of glucose monitoring, and the contribution of the proposed work. The design process, parametric analysis, and DBBPF structure are all covered in Sect. 2. Results and comments are presented in Sect. 3 with a model of a finger phantom. Results and discussion are presented in Sect. 4 with investigation on frequency shifts for finger phantom positions and glucose levels. Section 5 shows the machine learning of non-invasive blood glucose monitoring. Finally, the conclusion is outlined in Sect. 6.

### Design of microwave band pass filter sensor

**Fig. 2:**
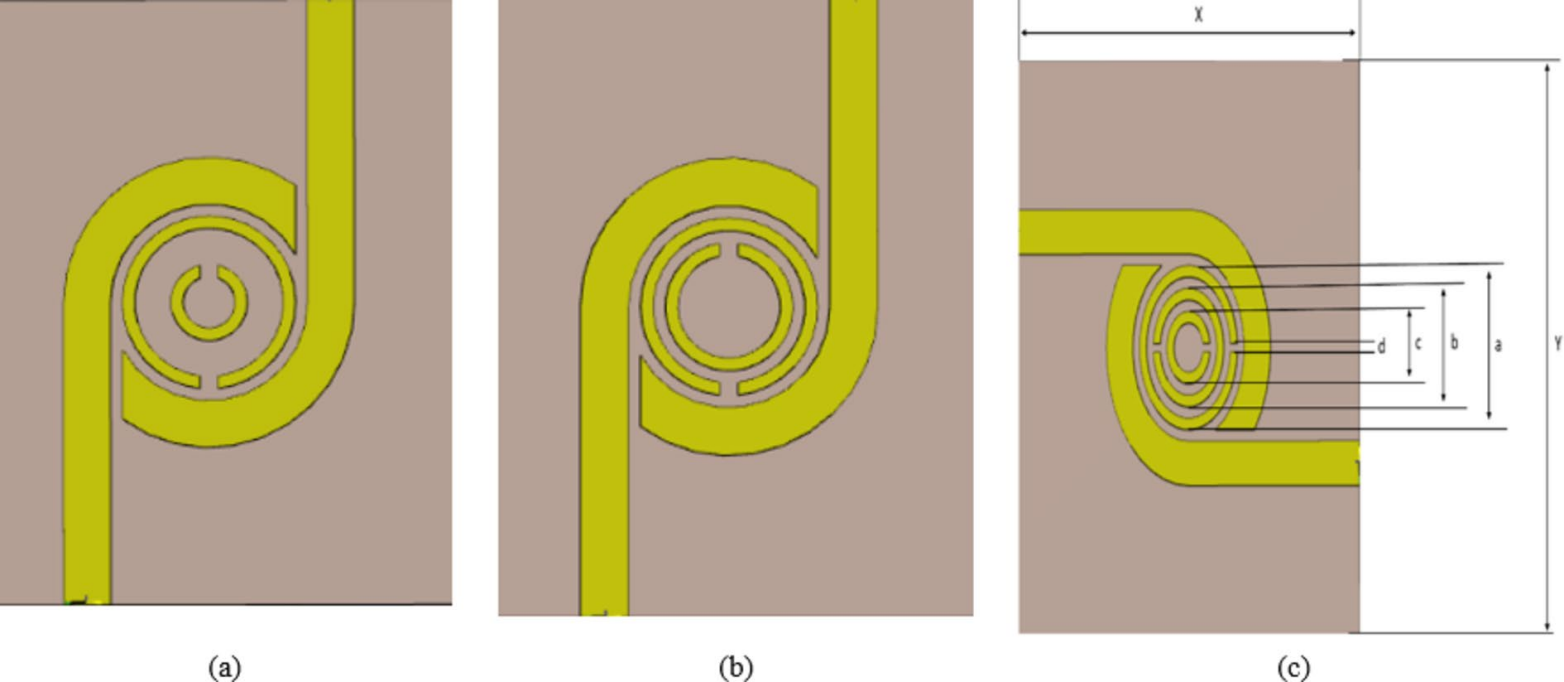
(**a**) First design 1,(**b**) Second design, and (**c**) Schematic of proposed sensor.

Changes in glucose concentration in aqueous solutions are known to cause modifications in the electrical properties of microwave biosensors. Therefore, the resonant frequency variations with the electrical properties of the material under test (MUT) form the basis of the co-design principle. Changes in glucose concentration in glucose/water solutions lead to changes in resonant frequency. Hence, this idea forms the basis for the proposed sensor design. In this section, a DBBPF is introduced and designed for use as a blood glucose sensor, as depicted in (Fig. 2). The filter is developed utilizing a three-dimensional electromagnetic simulator, specifically the computer simulation technology (CST) simulator. Additionally, an equivalent circuit model is generated later using the advanced design system (ADS).

Two 50 Ω microstrip transmission lines are intended to be connected to the resonator as feedlines as well. To match the connections, the characteristic impedance of 50 ohm is achieved by setting the width of the microstrip lines at 3.08 mm. The resonator sensor is constructed using FR4 epoxy substrate with relative permittivity ε_r_ = 4.4 and tan δ = 0.02. The usage of FR4 gives a lot of advantages as it is low cost and widespread in the market. The substrate’s overall dimensions are 40 × 40 × 1.6 mm^3^. It has been found that a distance of 0.8 mm between the SRR and the microstrip transmission line enhances coupling and boosts the sensitivity of the sensor.

This is attributed to the closer proximity between the transmission line and the resonator. Dual-band pass filter is designed with the targeted passband characteristics (the reflection and transmission characteristics) and SRRs are added within the DBBPF. Consequently, variation in resonant frequency is due to the change of the effective permittivity of the blood^35^. The initial design involves creating two concentric circular copper rings with a thickness of 35 μm and a gap of 2.4 mm to achieve resonance within the ISM Band, illustrated in (Fig. 2a). Subsequently, the gap is adjusted by reducing it to 0.8 mm, as depicted in (Fig. 2b).

This modification aims to enhance the sensitivity of the sensor to variations in glucose concentrations, particularly highlighting the effectiveness of split ring resonators (SRRs) in this context.

**Fig. 3:**
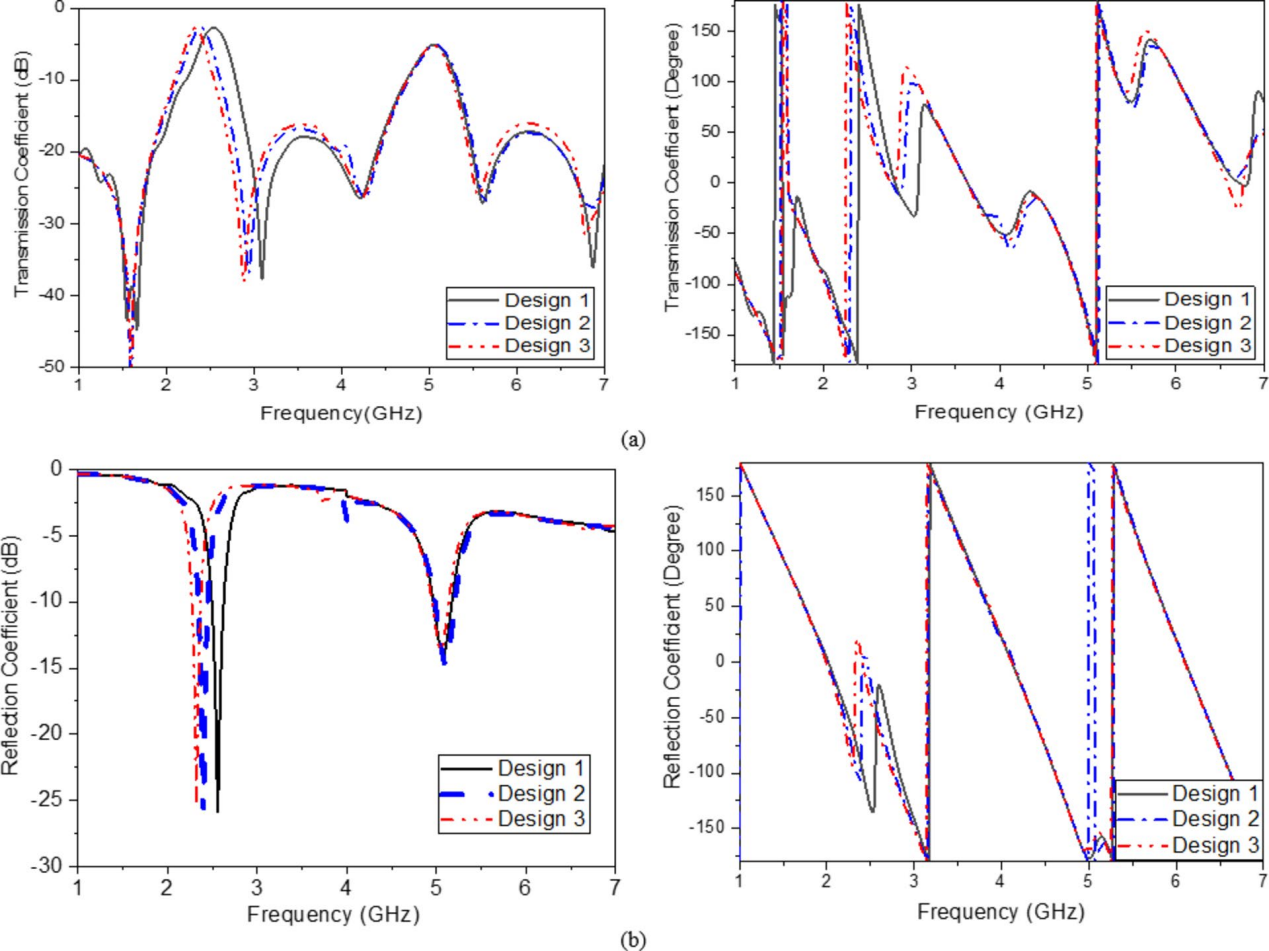
S-parameters for design steps; magnitude and phase (**a**) S_12_ and (**b**) S_11_.

**Fig. 4:**
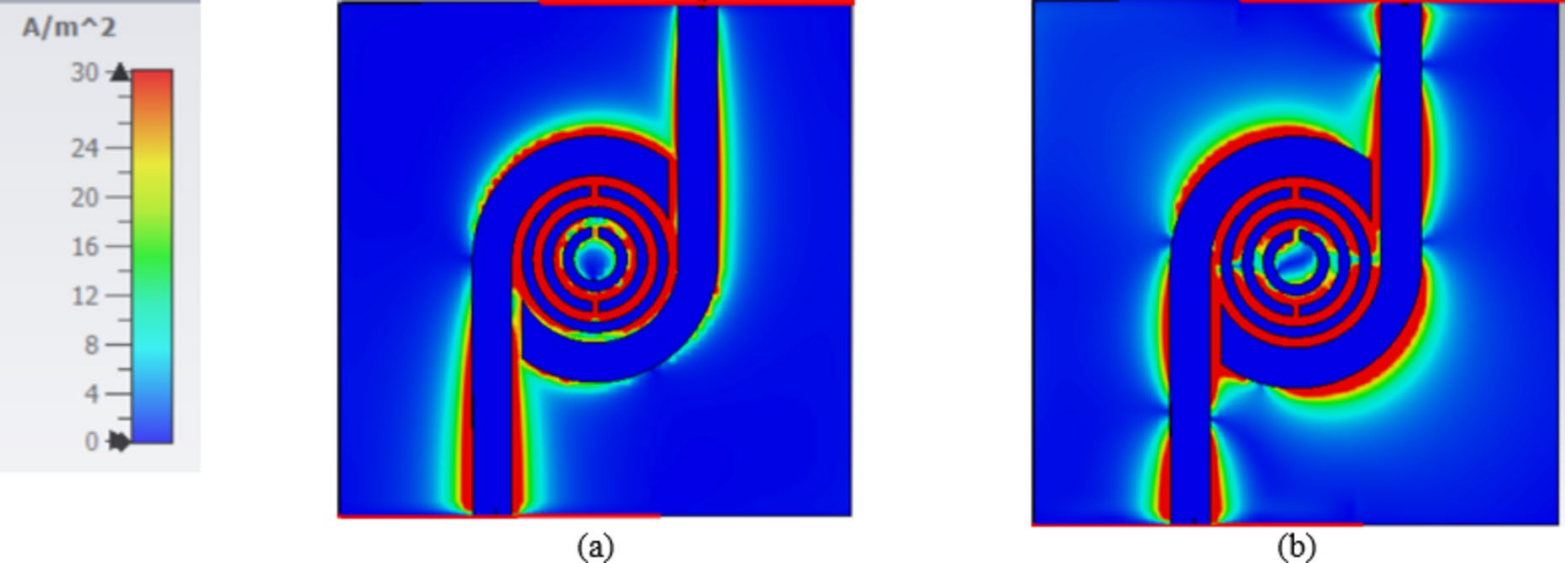
The current distribution of the BPF at two resonant frequencies at (**a**) 2.45 GHz and (**b**) at 5.2 GHz.

Finally, another small circle is added with a smaller size to create another SRR resonator to enhance sensitivity and resonate smoothly at the WLAN Band as shown in (Fig. 2c). The dimensions of the proposed sensor are shown in (Table 1). The results of the S-parameters (reflection and transmission coefficient) design steps are shown in (Fig. 3). Figure 4 displays the computed surface current distribution of the proposed filter at the two resonant frequencies 2.45 GHz and 5.2 GHz, respectively. The current distribution pattern indicates that the bulk of currents are located along the outer resonator ring for 2.45 GHz and the smaller resonator ring for 5.2 GHz, respectively, as well as over the 50 Ω transmission line. While the currents are concentrated around the split ring resonator cells as well as the transmission line.

**Table 1:** Dimensions of the proposed sensor (all dimensions in mm).

X	Y	a	b	c	d
40	40	11.44	8.24	5.04	0.6

### ADS circuit model

**Fig. 5:**
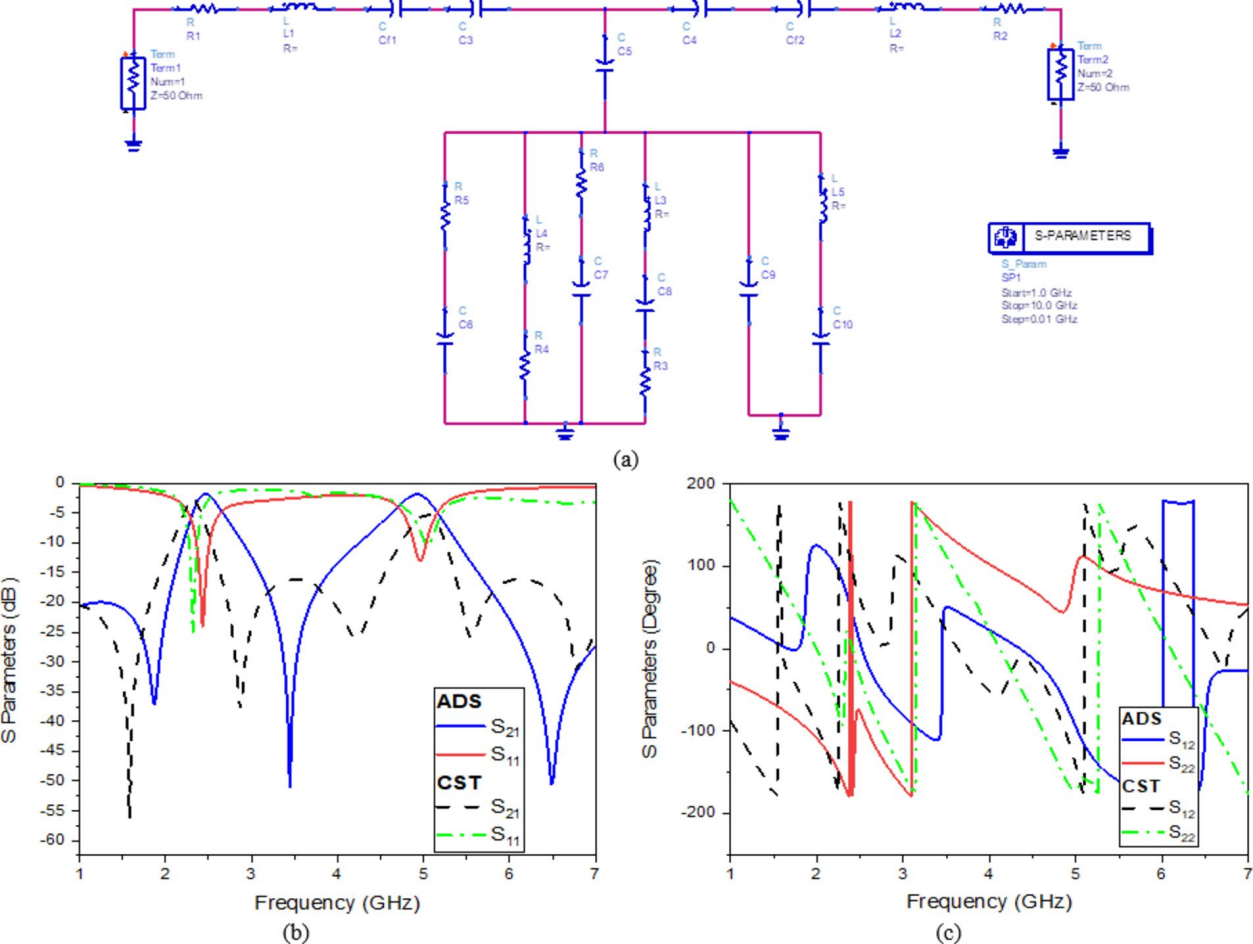
(**a**) The proposed sensor’s comparable circuit model, S parameters (**b**) Magnitude, and (**c**) Phase.

Furthermore, for the microwave DBBPF design, the circuit model is extracted using the advanced design system (ADS). The corresponding circuit, designed using ADS, is illustrated in (Fig. 5a). While all resonators share the same circuit topology, the values of the lumped parameters vary. The transmission lines are represented by capacitance *C*_*f1*_ and *C*_*f2*_, and the inductance *L*_*1*_ and *L*_*2*_.

Transmission line and substrate losses are computed by resistors *R*_*1*_ and *R*_*2*_, while the resonator circuit losses are measured by resistors *R*_*3*_, *R*_*4*_, *R*_*5*_, *R*_*6*_, *L*_*3*_, *L*_*4*_, *L*_*5*_, *C*_*5*_, *C*_*6*,_ C_8_, C_9,_ and *C*_*10*_ are the circuit parameters used to model the resonators are shown in (Table 2).

**Table 2:** Equivalent circuit model parameters.

*R*_1_	*R*_2_	*R*_3_	*R*_4_	*R*_5_	*R*_6_	L_1_	L_2_	L_3_	L_4_	L_5_
1.5 Ω	8 Ω	0.25Ω	0.225 Ω	0.5 Ω	0.5 Ω	1.79 nH	2.63 nH	0.74 nH	0.52 nH	1.44 nH

***C***_***f1***_	***C***_***f2***_	***C***_***3***_	***C***_***4***_	***C***_***5***_	***C***_***6***_	***C***_***7***_	***C***_***8***_	***C***_***9***_	***C***_***10***_	***TL***
2.1 pF	2.1 pF	10 pF	2.88 pF	8.46 pF	1.26 pF	0.01 pF	0.91 pF	0.01 pF	1.86 pF	50 Ω

As demonstrated in (Fig. 5b, c), there is a good agreement between the simulation and the S-parameters magnitude and phase, respectively obtained from the equivalent circuit represented by the SRR sensor. Good agreement between the electromagnetic simulator and ADS circuit. Differences in results could be attributed to non-inclusion of the mutual coupling between the circuit inductor elements as well as the fringing effect loss in circuit models.

### Proposed technique of glucose levels

The effect of glucose solution concentration on the dielectric properties is studied in this section. To obtain a rough estimate of the true glucose values in human blood. Glucose samples are generated at different glucose concentrations of 50, 100, 200, and 400 mg/dL by using two scenarios. The first scenario was done by using a container filled with different glucose samples, and the second scenario was done by using the human figure phantom model and changing the blood glucose level. The specific layer that accumulates varying glucose concentrations at varying frequencies is known as the blood layer.

#### First scenario

**Fig. 6:**
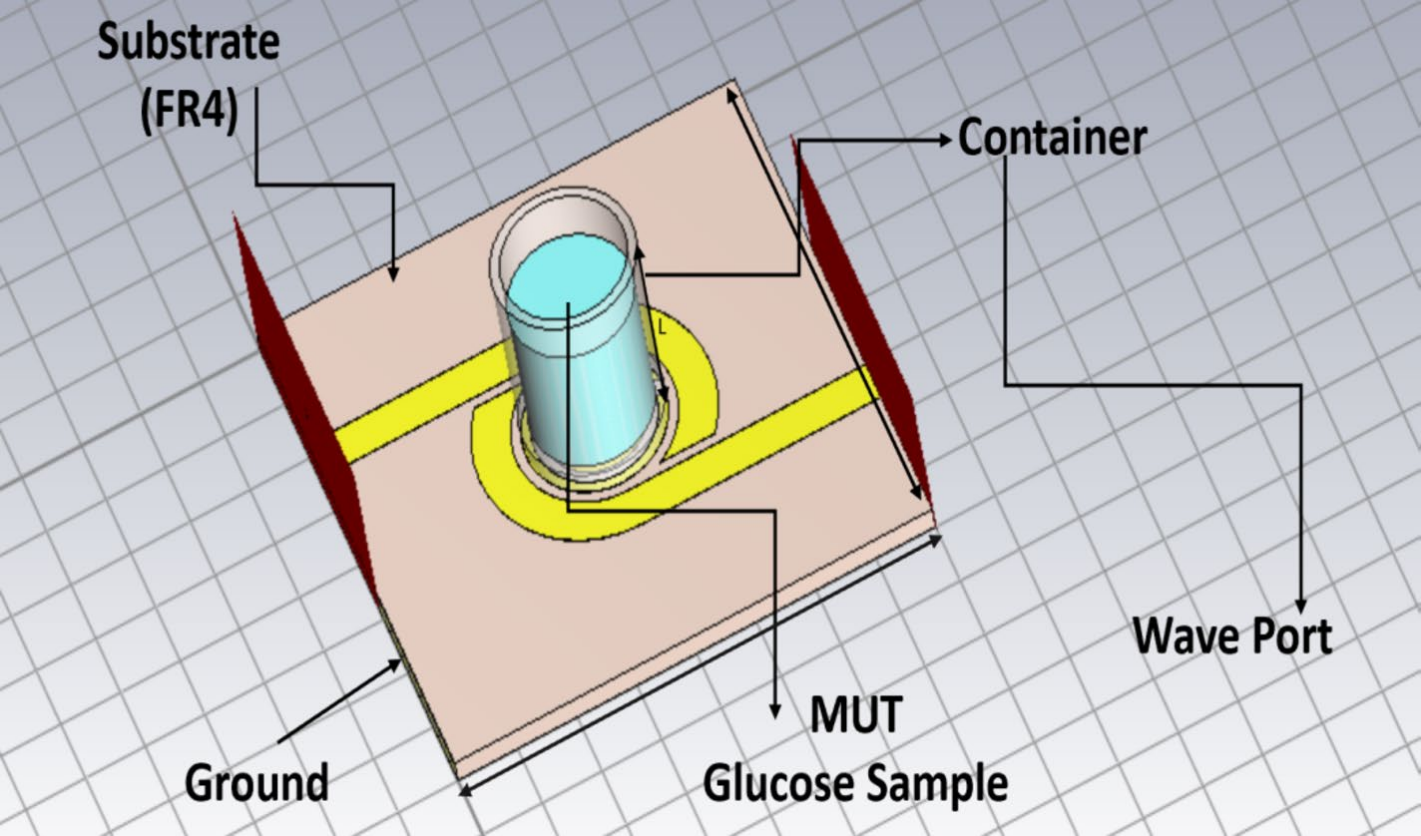
Model of the proposed filter with the container used for loading glucose sample.

**Fig. 7:**
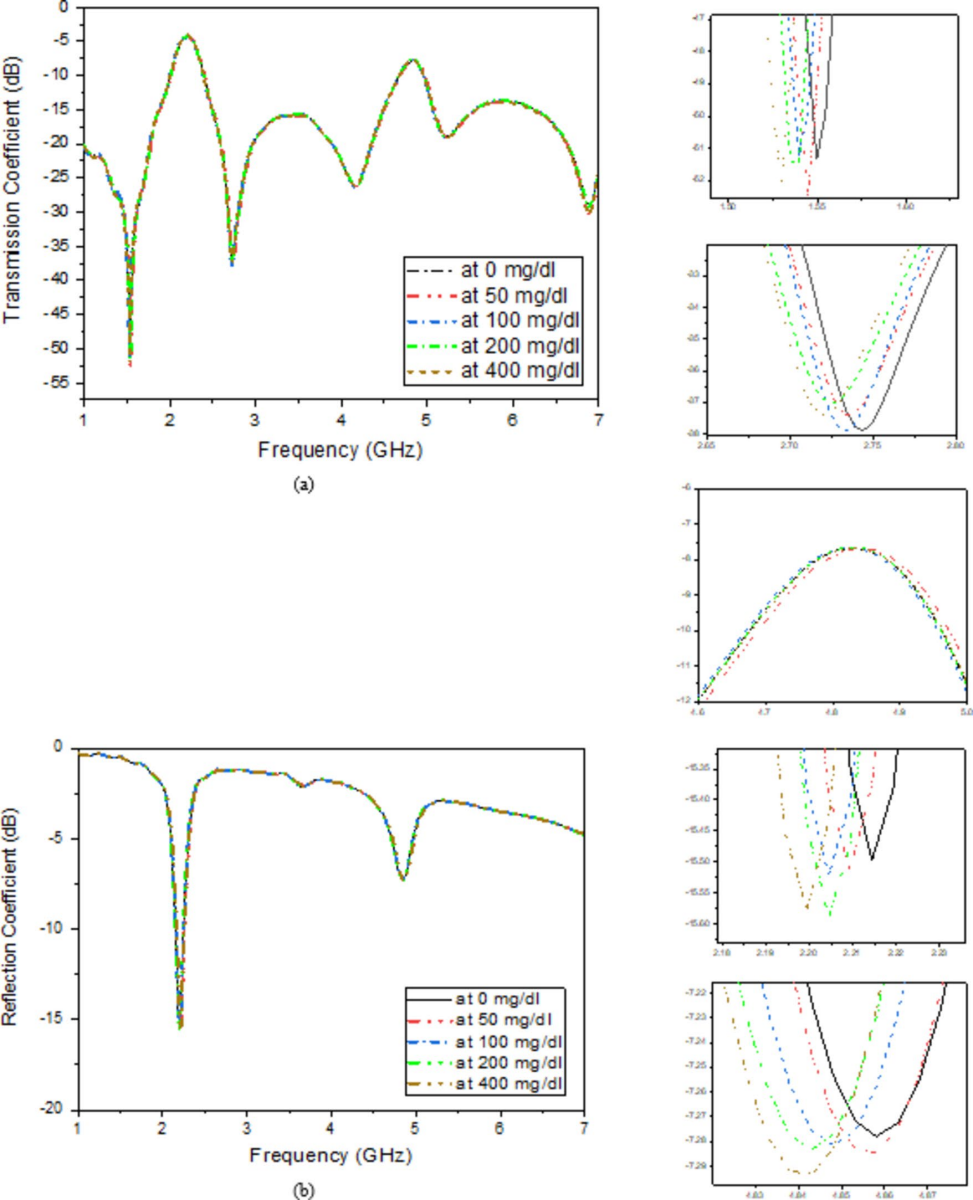
Simulated (**a**) Transmission coefficient with different glucose concentrations and (**b**) Reflection coefficient with different glucose concentrations.

For the best results with the fewest losses and responsiveness, the container should be covering the whole SRR as shown in (Fig. 6). The container holding sample under test (SUT) is made of polypropylene with dielectric properties of *ε*_*r*_ *= 2.2*,* tan δ = 0.0001* and thickness h = 1.5 mm. The thickness of the base of the container is 1.5 mm, the diameter is 11.44 mm and the height of the container is 15 mm as shown in (Fig. 6). When glucose samples are tested at different concentrations in a container above the SRR circuit, the transmission and reflection parameters are recorded at different frequencies. To display the glucose content, the dielectric permittivity ε and the material’s conductivity σ are also considered as shown later in the next section. The incorporation of the SRR in the proposed design and sensing problem is related to its ability to acquire higher sensitivity and high-quality factor resonance peaks. Aqueous glucose solutions are measured in vitro using the proposed DBBPF as shown in (Fig. 7). To differentiate between glucose samples, a high Q-factor sensor is proposed. Tables 3 and 4 illustrate the transmission and reflection coefficient, respectivel, y subjected to glucose solutions with different concentrations in the range of 50–400 mg/dl.

**Table 3:** Simulation results of transmission coefficient of frequency and phase shift of the proposed sensor.

∆F (MHz)	50 mg/dl	100 mg/dl	200 mg/dl	400 mg/dl
1st resonance	9.979	0.002	9.939	9.943
2nd resonance	7.396	0.009	0.297	7.072
Phase shift (degree)	0.9298	−0.000136	−0.1889	−1.2429

**Table 4:** Simulation results of reflection coefficient of frequency and phase shift of the proposed sensor.

∆F (MHz)	50 mg/dl	100 mg/dl	200 mg/dl	400 mg/dl
1st resonance	9.956	0.033	0.064	9.914
2nd resonance	14.77	0.0022	0.087	14.753
Phase shift (degree)	0.3725	−0.007369	−0.02726	0.3687

#### Second scenario

**Fig. 8:**
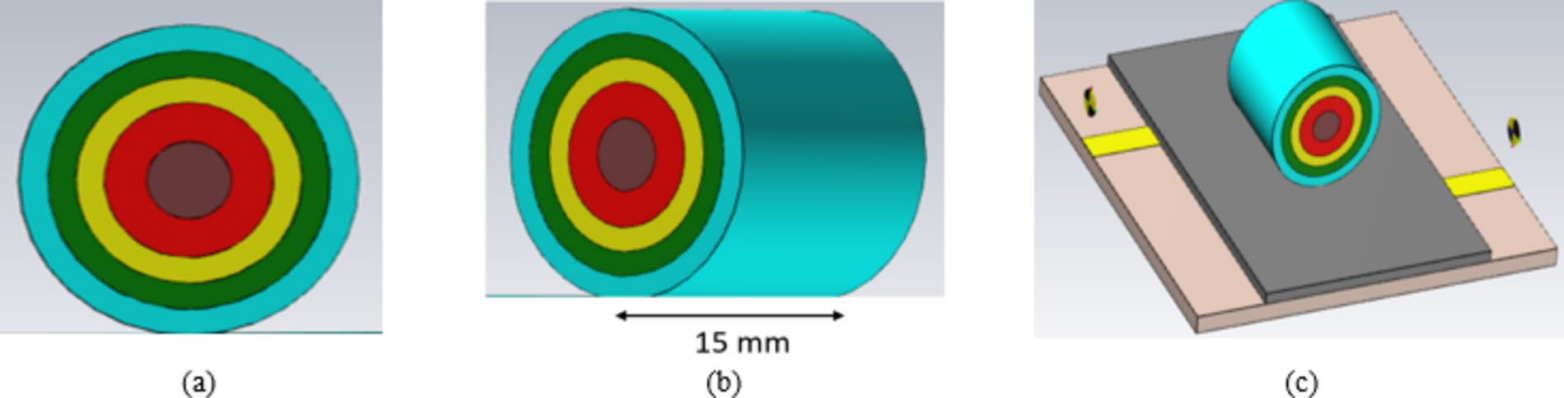
Model of a finger phantom (**a**) Elevation view, (**b**) 3D view, (**b**) 3D and (**c**) Phantom on the top of the proposed filter.

The creation of a sensor that can track changes in blood glucose concentration from the fingertips is the main goal of this section. To achieve this, a finger phantom model is built that replicates the real human as illustrated in (Fig. 8). The Debye model and the Cole-Cole model are the two models that are typically utilized in biomedical applications for modeling body tissues^38^. Since the Cole-Cole model yields a more precise values for the tissues’ dielectric characteristics, it is often favored. For the finger phantom model, the Cole-Cole model is employed, with each layer’s frequency-dependent features based on the frequency utilized, as indicated in (Table 5)^38^.

1 $$\:{\epsilon\:}^{*}\left(\omega\:\right)={\epsilon\:}^{{\prime\:}}-j{\epsilon\:{\prime\:}}^{{\prime\:}}=\:{\epsilon\:}_{\infty\:}+\frac{{\epsilon\:}_{s}-{\epsilon\:}_{\infty\:}}{1+{\left(j\omega\:\tau\:\right)}^{\left(1-\alpha\:\right)}}+\frac{{\sigma\:}_{s}}{j\omega\:{\epsilon\:}_{o}}$$

**Table 5:** Dielectric properties and the conductivity at different glucose concentrations and frequencies.

Glucose concentration (mg/dL)	Frequency (GHz)	*ε*_*r*_	$\:\varvec{\sigma\:}\:$(S/m)
50	2.45	69.74	3.498
100		69.7	3.482
200		69.67	3.47
400		69.59	3.441
50	5	64.62	7.078
100		64.56	7.069
200		64.51	7.062
400		64.39	7.046

where $\:{\epsilon\:}^{*}$, ε′ and ε′′ are the dielectric constant and dielectric loss factor values of a material, respectively, ε_s_ is the static permittivity, ε_∞_ is the permittivity at infinite frequency,* ω* is the angular frequency,* τ* is the relaxation time,* σ* is the static conductance, ε_0_ is the permittivity in free space,* α* is the exponent parameter. The electrical properties of materials with varied dielectric constants and conductivities are used to simulate different layers of the finger, including skin, fat, muscle, blood, and bone, as shown in (Table 6).

**Fig. 9:**
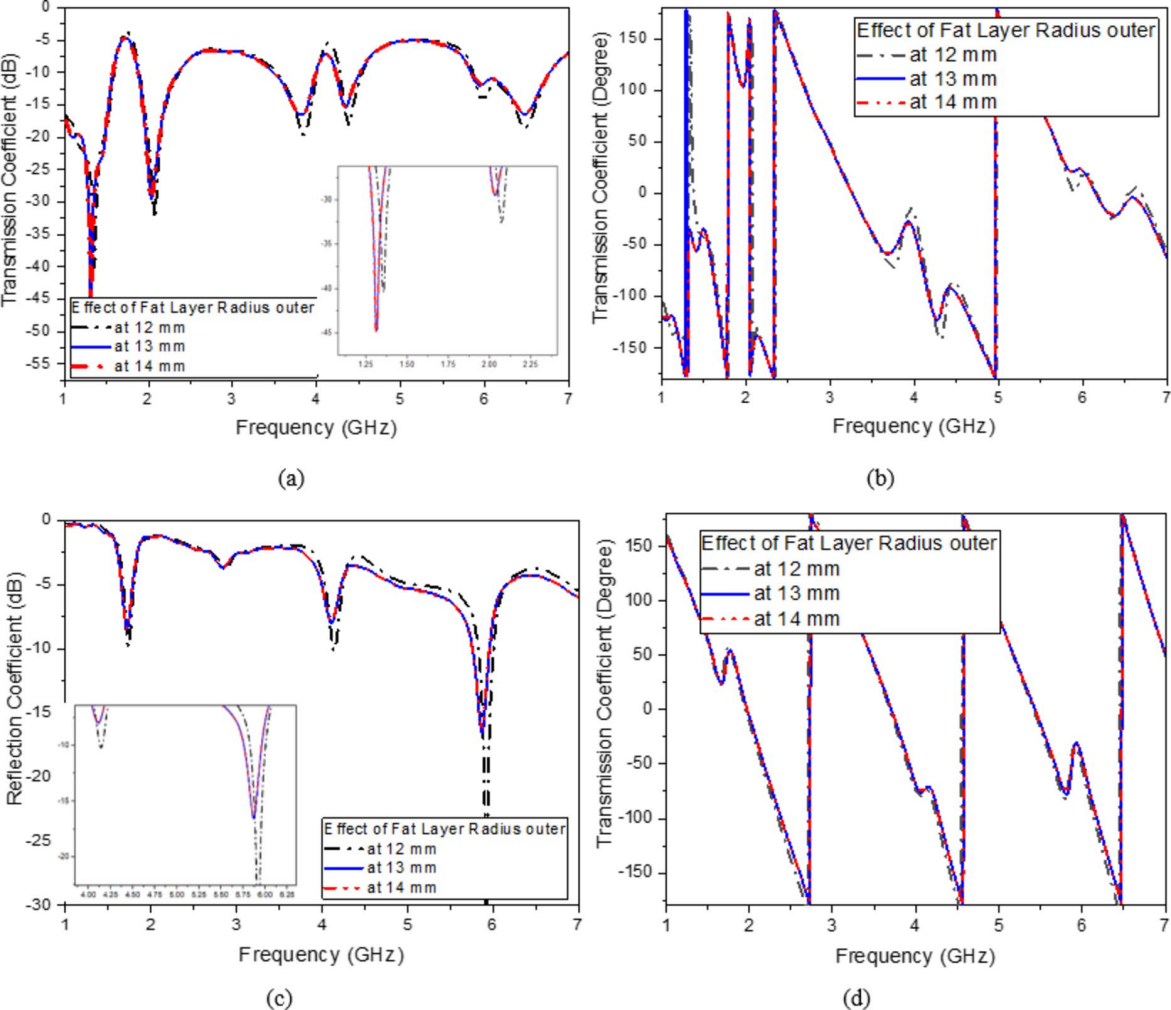

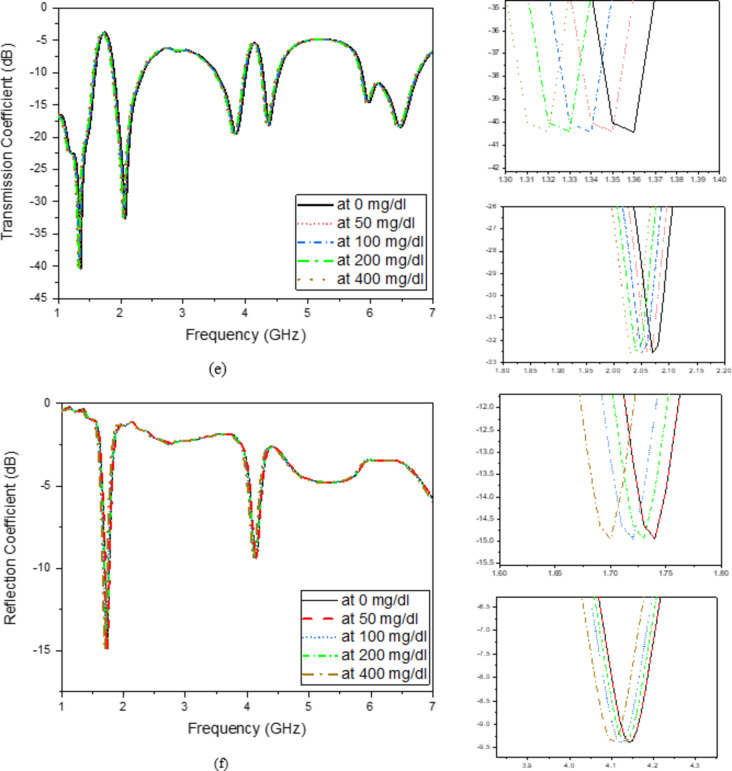
Effect of finger size (**a**) Transmission magnitude, (**b**) Transmission phase, (**c**) Reflection magnitude, (**c**) Reflection phase, (**d**) Simulated transmission coefficient with a finger on the top loaded with different glucose concentrations and (**e**) Simulated reflection coefficient with a finger on the top loaded with different glucose concentrations.

As illustrated in (Fig. 8c), the finger phantom is positioned on the top of a glass buffer with the thickness of 1 mm over the proposed filter. Figure 9 shows the appropriate shifts of resonant frequency and magnitude. In this instance, variations in blood concentrations are responsible for the observed shifts in frequencies. Therefore, for the glucose change from 0 to 400 mg/dL, a growing or decreasing frequency shift is seen. Figure 9a, b depict the S_11_/S_21_ characteristics and how they vary for varying glucose concentrations. As a result of increased field contrast, it makes the sensor suitable for glucose monitoring. Better values of frequency shift can be produced when there are more confined in the electric field around the human finger due to the presence of SRR cells.

**Table 6:**
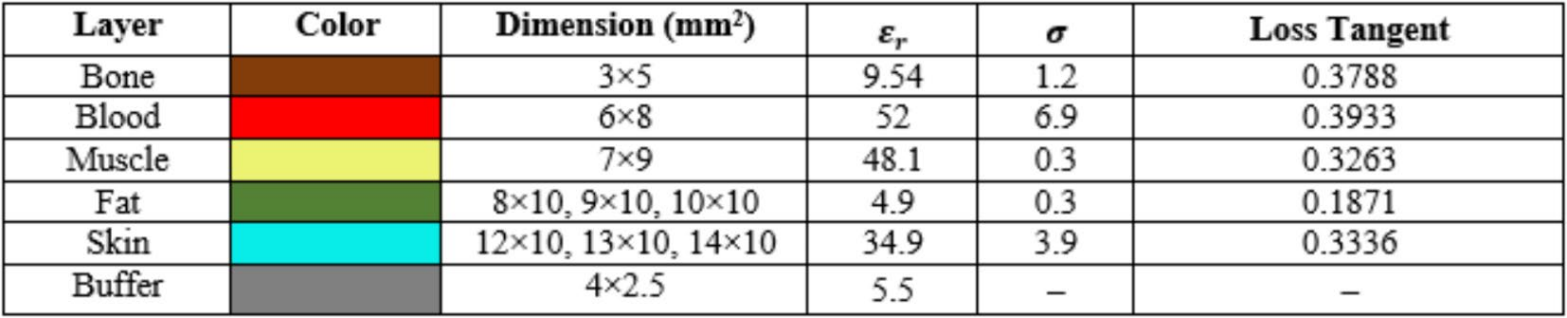
Description of the layers of the finger phantom.

**Fig. 10:**
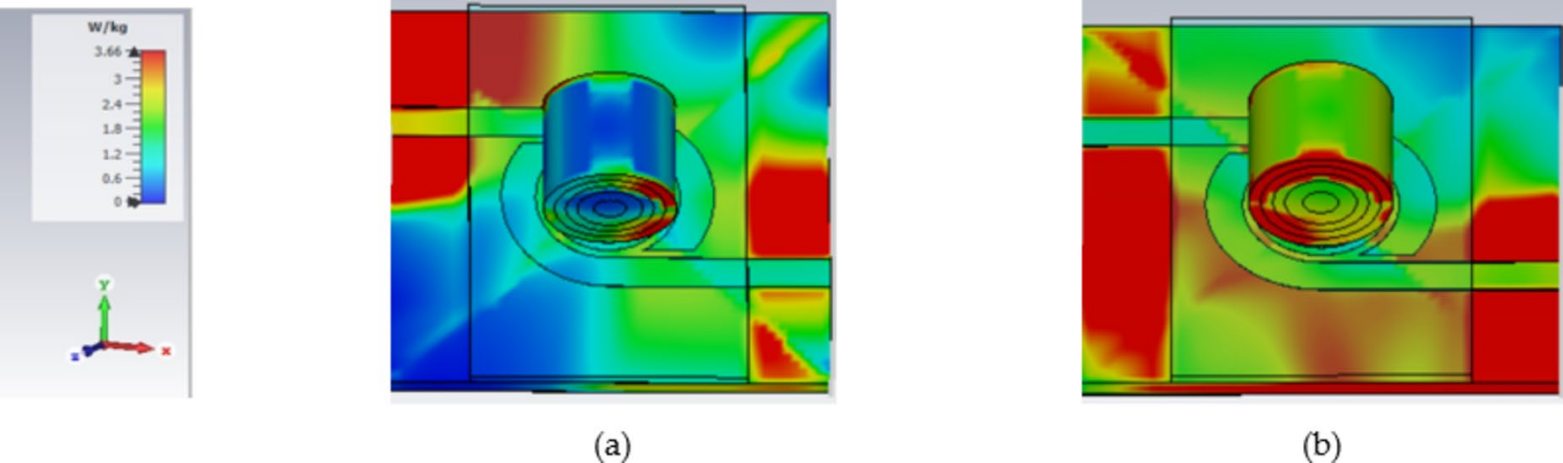
SAR Simulation of proposed DBBPF with a human finger over 1 g of tissue-based at (**a**) 2.45 GHz and (**b**) 5.2 GHz.

Finger size affects the accuracy of non-invasive blood glucose monitoring with microwave sensors depending on how the sensor’s electromagnetic fields interact with tissue. Larger fingers, with more tissue mass, influence microwave signal propagation, leading to differences in signal absorption and reflection, which can distort glucose readings. To improve accuracy, sensors might require calibration for varying finger sizes, or they could incorporate adaptive designs and advanced signal processing or machine learning techniques to account for tissue volume differences among users. As shown in Fig. 9, changes in finger size cause slight variations in the phase or magnitude of the S-parameters. While these variations are within a reasonable range, the housing design effectively mitigates this issue. The thickness of the skin and fat layers varies from 12 to 14 mm and 8 to 10 mm, respectively, in 1 mm increments, as shown in (Fig. 9a–d). The resulting frequency shift (*∆f*) of the proposed sensor is also illustrated in (Fig. 9). It is evident that the resonant frequency decreases linearly as skin thickness increases until the finger fully covers the sensor shape, after which the frequency shift saturates, as shown in (Fig. 9a–d). This variation in dielectric values causes variation in the electric field. Figure 9e, f illustrate varying amounts of glucose with resonates, respectively, at 2.45 and 5.2 GHz. Individuals with diabetes should typically not be exposed to health risks, so the sensor should be the low-radiation microwave. In addition, safety must be tested, and the value of humans must be calculated, even if it is not used continuously. The specific absorption rate, or SAR, can be used to determine how much radiation is absorbed by human tissues, as shown in (Fig. 8). The simulated SAR values at the resonant frequency at 1 g and 10 g of tissue based on the IEEE C95.3 standard for a device are 1.6 and 2 W/Kg respectively. The proposed filter realizes SAR values of 1.7 and 1.2 W/Kg at 2.45 GHz and 2.1 and 1.5 W/Kg, respectively, at 5.2 GHz at 0 dBm, as shown in (Fig. 10).

### Fabrication, characterizations, and measurements

**Fig. 11:**
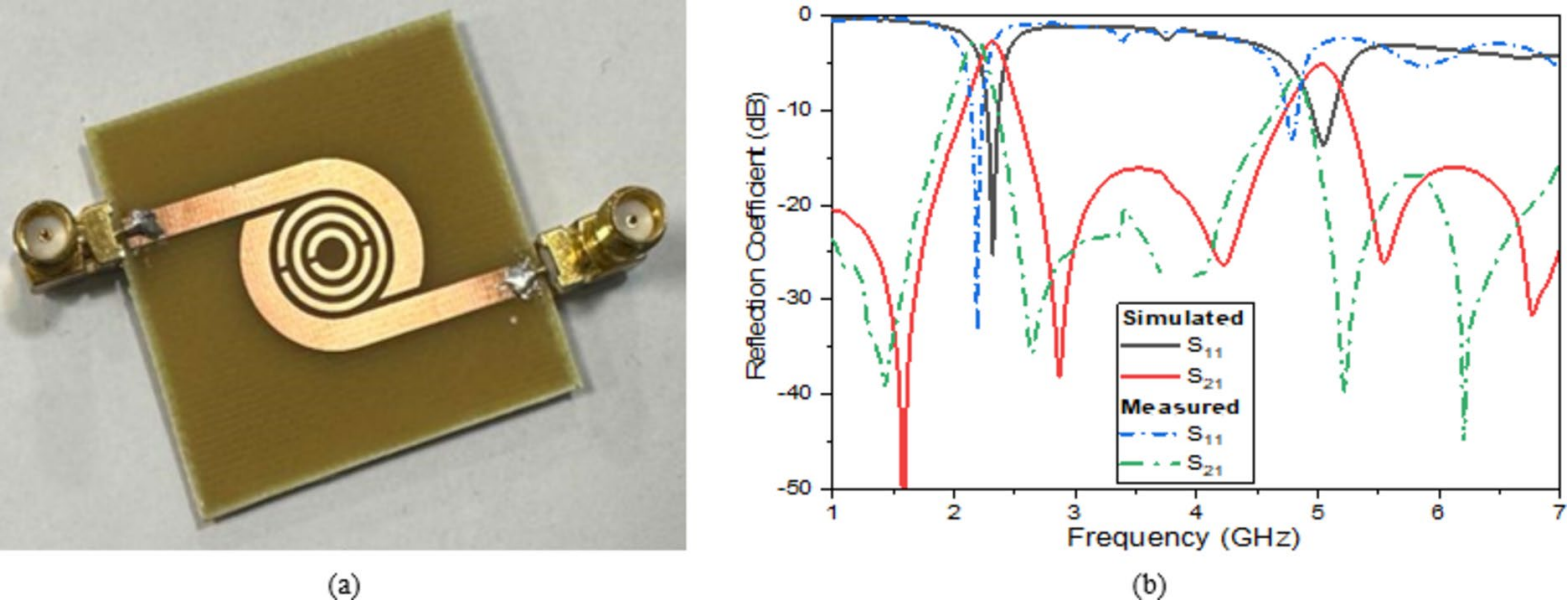
(**a**) The fabricated proposed DBBPF sensor and (**b**) Simulated and measured values of S-parameter.

The proposed dual bandpass filter sensor is fabricated, as shown in (Fig. 11a), to validate the design results of the proposed DBBPF sensor, as shown in (Fig. 11b). The sensor is fabricated in the Microstrip department of the Electronics Research Institute by using the photographic technique to fabricate the Printed circuit board (PCB), as shown in (Fig. 11).

#### Sensor on air

Before inserting any glucose samples throughout the frequency range of operation 1–7 GHz frequency band, the fabricated sensor is assessed using the Rohde & Schwarz ZVA 67 Network Analyzer. Figure 11b displays the measured and simulated S-parameter of the unloaded sensor. The simulated minimum reflection coefficient at 2.4 GHz is −25 dB, and the measured minimum reflection coefficient at 2.3 GHz is −33 dB. At 1.5 GHz, the measured minimum transmission coefficient is -39.3 dB, while the simulated minimum Transmission Coefficient is – 56 dB. There is good agreement between the simulated and measured results, which verifies the simulation model for the proposed sensor. Several possible explanations exist for the discrepancies between both results at different frequencies. First, the impact of the coaxial wire utilized for the measurement. Second, the SMA solder was utilized to join the SMA conductor to the transmission line. Third, manufacturing and measurement tolerances in the filter’s placement, additional electromagnetic interference signals in the environment, and the best model to employ for simulation.

#### SAR measurement

The specific absorption rate, or SAR, can be used to determine the quantity of radiation that is absorbed by human tissues. The sensor used with human contact could be deemed safe if its maximum SAR value is less than a certain value as given above. Diabetic individuals should typically not be exposed to any health dangers when using low-radiation microwaves.

Therefore, a dedicated instrument constructed at different power levels for the sensor is used to conduct SAR measurements in the central laboratories of the Electronics Research Institute by terminating the other filter port by 50 Ω. Table 7 shows the measured SAR levels for the proposed sensor at 2.45 and 5.2 GHz.

**Table 7:** Comparison of measured SAR levels for dual bandpass filter sensor.


Power (dBm)	2.45 GHz		5.2 GHz	
SAR evaluate	1 g	10 g	1 g	10 g
0	0.005	0.002	0.08	0.023
5	0.009	0.01	0.097	0.023
10	0.05	0.02	0.091	0.022
15	0.06	0.04	0.063	0.016
20	0.093	0.071	0.08	0.02

### Glucose characterization

**Fig. 12:**
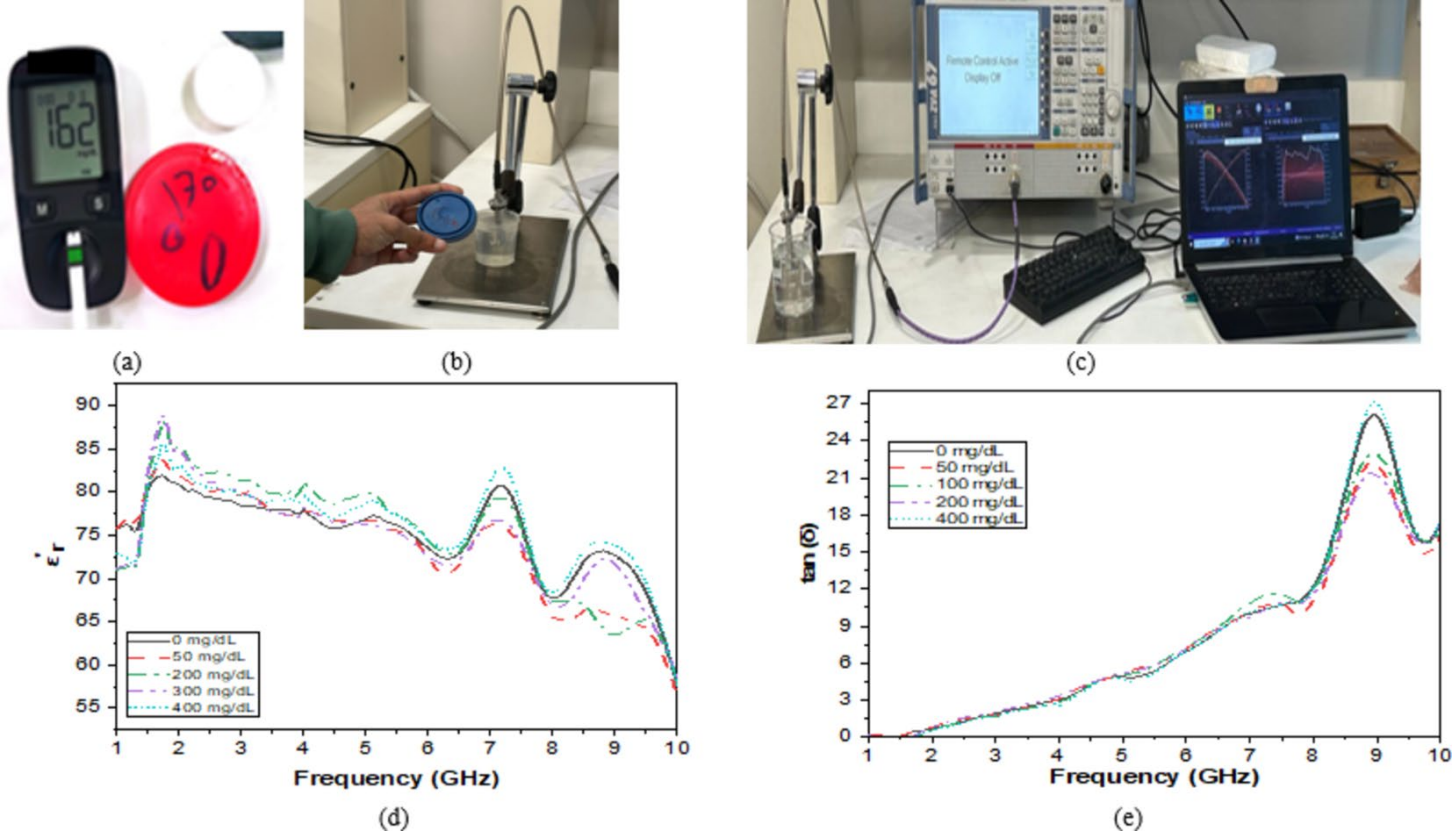
(**a**) Measurement using Glucometer for prepared sample, (**b**) Characterization of samples by using DAK, (**c**) DAK setup of samples, Dielectric properties for the prepared glucose samples: (**d**) Relative permittivity $\:{\epsilon\:}_{r},$ and (**e**) Loss tangent tan (δ).

First, the impact of glucose solution concentration on the electrical properties characteristics is investigated. To obtain an approximation of the true values of the human blood glucose, the glucose solution samples are made by dissolving glucose granules in de-ionized water. Different glucose concentrations of 0, 50, 100, 200, and 400 mg/dl are prepared as shown in (Fig. 12a).

Using a glucometer (Accu-Chek Active), the sample concentration is assessed as shown in (Fig. 12b), and the complete setup of the Dielectric Assessment kit (DAK)^39^ is shown in (Fig. 12b). The measurement verifies that it is necessary to obtain the required concentrations with a tolerable degree of accuracy. The measurement results are displayed in (Fig. 12c) by using a VNA, the electrical properties parameters of the samples (i.e., dielectric constant real and imaginary) within the frequency range of 1–10 GHz are measured as shown in (Fig. 12d, e). The results of the experiments show that when the variation of the concentration increased, the change of the dielectric constant increased.

#### Sensor loaded with glucose concentrations

An ethical approval from the National Institute of Laser Enhanced Science, Cairo University with number NILES-EC-CU 24/1/2, date of issuing is January 8, 2024, has been obtained before conducting testing with volunteers and by the Helsinki declaration.

#### Measured first scenario

**Fig. 13:**
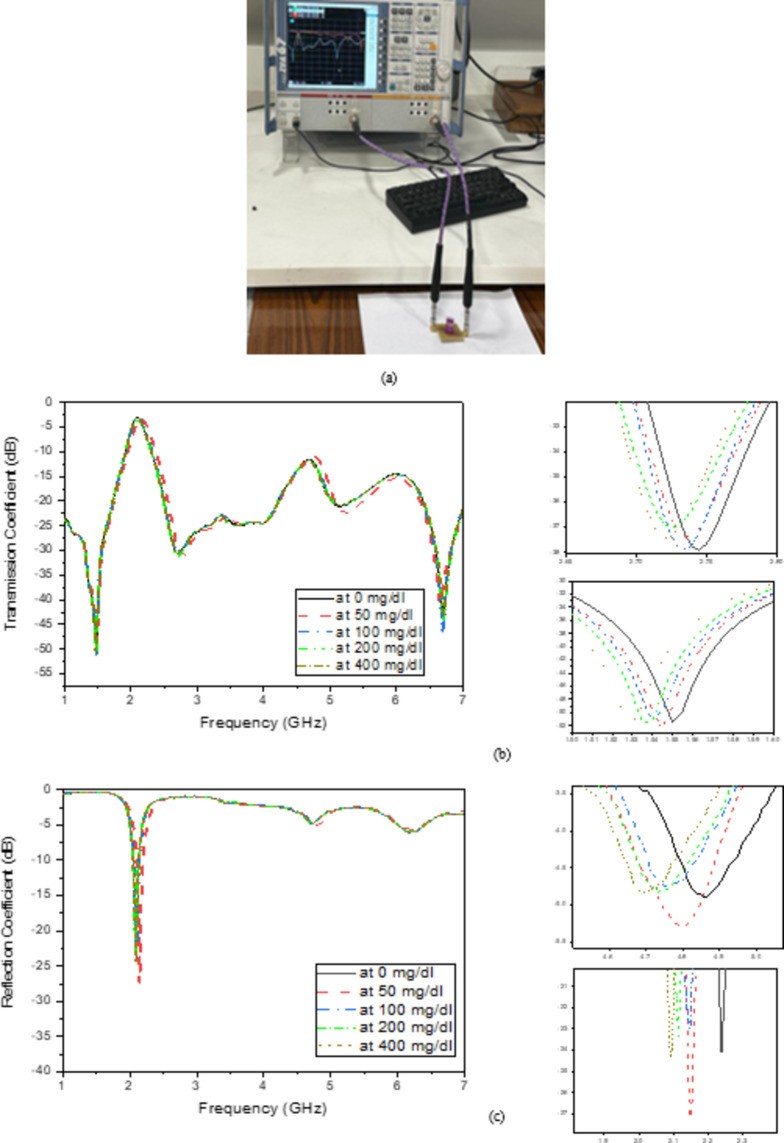
(**a**) Proposed filter sensor loaded with glucose concentration, measured of sensor loaded with different glucose concentrations (**b**) transmission coefficient, and (**c**) reflection coefficient.

The performance of the proposed microwave filter sensors will be presented in this section with a polymer container attached to the proposed sensor. The container is symmetrically positioned over the SRR cell with the dimensions 11.44 × 2 × 1 mm^3^. The container is filled with the same predetermined amount of glucose concentration, and a Rohde & Schwarz VNA is employed, as depicted in (Fig. 13a). Various glucose concentrations of 0, 50, 100, 200, and 400 mg/dL are then added using a pipette to the container placed above the sensor with the same volume for each Concentration. The results depicting the reflection and transmission coefficients for the different concentrations are presented in (Fig. 13).

#### Measured second scenario

The second scenario is conducted by using fingertips to validate the theory. Fingertip is a good option for measuring blood glucose levels, as it is close to the veins that carry blood from the human. All experiments involving human subjects and/or tissue samples were conducted by the ethical approval standards of the NILES Ethics Committee at National Institute of Laser Enhanced Sciences, Cairo University (approval number: NILES-EC-CU 24/1/2) on January 8, 202. The proposed study was carried out in accordance with the Helsinki declaration. Informed consent was obtained from all participants and/or their legal guardians before their inclusion in the study.

**Fig. 14:**
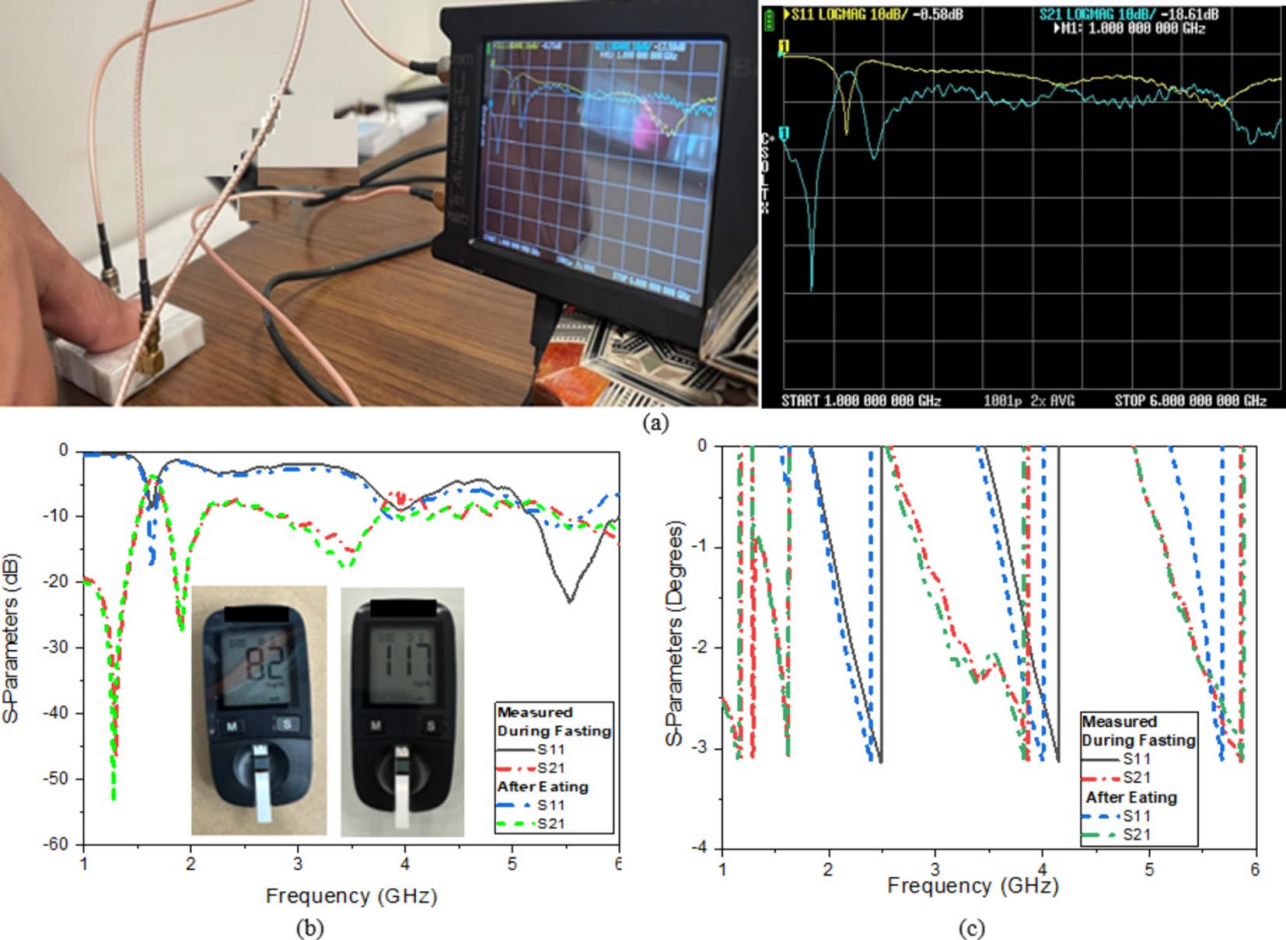
(**a**) Measurement setup, S parameters, and glucometer reading before fasting and after fasting (**b**) Magnitude and (**c**) Phase.

The first step of examinations is conducted, it is necessary to ask volunteers to fast for six hours following their last meal before the test. The volunteers were allowed to rest for around fifteen minutes until their bodies stabilized before the test started and the volunteer was instructed to wash his hands to get rid of any pollutants before they were evaluated at 25^◦^C room temperature by using a nano-vector network analyzer from 0.5 GHz to 6 GHz as shown in (Fig. 14a). The commercial standard method ACCU-CHEK ACTIVE is used to do the intrusive measurements as shown in (Fig. 14b, c). The result for the volunteer before fasting was 82 mg/dl and 117 mg/dl after fasting. The sensor’s resonance frequency shifts in response to changes in blood glucose levels as shown in (Fig. 14b, c).

## Discussion

This paper provides evidence for using dual BPF in vitro experiments to distinguish between different glucose concentrations in diabetic patients with hypoglycemia, normoglycemia, and hyperglycemia. The key concept relies on the low-cost FR4 substrate’s absorption of various glucose/water solutions on the filter sensor region. Therefore, the fluctuation in dielectric characteristics of different glucose concentrations inside the detecting region causes the filter-based sensor’s resonance frequency to fluctuate. The frequency detection resolution (FDR) is a performance measure for glucose concentration detection sensors. In /(/), the FDR can be taken out as^44^.

2 $$\:FDR=\frac{\varDelta\:F}{\varDelta\:C}\:$$

For detecting the sensor, as an example, glucose level is varied from 50 to 100 mg/dl, resulting ∆C of 50 mg/dl and ∆F being equal to 101.3 MHz, so the FDR is 2.026 MHz/(mg/dl).

The sensor’s accuracy, as stated in Eq. (3), is ascertained by its sensitivity^44^.

3 $$\:{S}_{n}=\frac{FDR}{{F}_{fl}}\:\times\:100$$

Where FDR is 2.026 MHz/(mg/dl) and *F*_*FL*_ the sensor’s resonance frequency for freeload is 5.1 GHz. Thus, the sensitivity of the sensor is 0.0397 1/(mg/dL).

Sensitivity, which may be computed as the ratio of the change in the magnitude of S_11_/S_21_, is a crucial component in assessing a sensor’s reaction capability. The sensitivity of the sensor is improved with a higher ratio. A high ratio suggests that the sensor is extremely sensitive and capable of picking up on even minute variations in the concentration of glucose. That is expressed in Eq. (4.1) and Eq. (4.2)^44^ .

4.1 $$\:Sensitivity=\frac{\varDelta\:{\text{S}}_{21}}{\varDelta\:\text{c}}$$

4.2 $$\:Sensitivity=\frac{\varDelta\:S11}{\varDelta\:c}$$

By measuring $\:\text{i}\text{n}\text{c}\text{r}\text{e}\text{m}\text{e}\text{n}\text{t}11$ it is equal to 0.5506 dB and $\:\varDelta\:c$ is the difference between glucose levels equal to 50 mg/dL. Thus, the sensitivity is 0.011 dB/(mg/dl). All S_11_ and S_21_ results for Low and High resonant Frequency of the DBBPF are shown in (Table 8). The data obtained with the created microwave filter sensor is contrasted with other advances in (Table 9). We chose the results where the transmitted signal and the reflected signal were both utilized as the measured parameters so that the findings of this study could be compared to those of other studies.

**Table 8:** Sensitivity values in low/high resonant frequency of DBBPF.

	Low frequency			High frequency		
	Equation (2) MHz/(mg/dL)	Equation (3) 1/(mg/dL)	Equation (4) dB/(mg/dL)	Equation (2) MHz/(mg/dL)	Equation (3) 1/(mg/dL)	Equations (4.1) & (4.2) dB/(mg/dL)
S_11_	0.9	0.038	0.08943	2.026	0.0397	0.011
S_21_	0.9	0.038	0.0013	1.35	0.026	0.0083

**Table 9:** Comparison between proposed sensors with other published sensors in literature.

Ref.	Concentrations (mg/dL)	Frequency GHz	S$\left( {\frac{{\user2{MHz}}}{{\user2{mg}/\user2{dL}}}} \right)$	S_*n*_$\:\left(\frac{1}{\varvec{m}\varvec{g}/\varvec{d}\varvec{L}}\right)$	Size MM^2^	MUT	Machine learning
^45^	0–300	2-2.5	NM	0.003	NM	A.G	No
^46^	0–1800	4.4725	0.0039	8.7 × 10^− 5^	22 × 12	A.G	No
^47^	75–150	8.8	1.2	1.7 × 10^− 2^	52 × 24	A.G	No
^48^	18–540	1.156	7.5 × 10^− 5^	6.48 × 10^− 6^	25 × 25	A.G	No
^49^	0–5000	4.18	0.026	6.2 × 10^− 4^	50 × 20	A.G	No
DBBPF	0–400	2.45/5.2	0.9	0.038	40 × 40	A.G/finger	Yes

*NM* not mentioned, *MUT* material under test. *A.G* aqueous glucose.

The proposed sensor excels in terms of sensitivity and offers a balance between compactness and dual-frequency operation, making it more versatile for glucose sensing applications, and it shows significantly higher sensitivity compared to other sensors: Frequency Sensitivity (S) = 0.9 MHz/(mg/dL) (much higher than others like Ref^47^. at 0.0039, Ref^48^. at 1.2). Numerical Sensitivity (Sn) = 0.038 1/(mg/dL) (higher than others like Ref^50^. at 0.00062 or Ref^47^. at 8.7 × 10⁻⁵). It operates at dual frequencies (2.45/5.2 GHz), which enhances its performance across a broader range of concentrations (0-400 mg/dL) compared to some other sensors that operate in narrower frequency ranges (e.g., 1.156 GHz in Ref^49^. or 8.8 GHz in Ref^48^). Though not the smallest, its size (40 × 40 mm²) is relatively compact given its higher sensitivity, compared to larger sensors like 52 × 24 mm² (Ref^48^). or 50 × 20 mm² (Ref^50^).

**Fig. 15:**
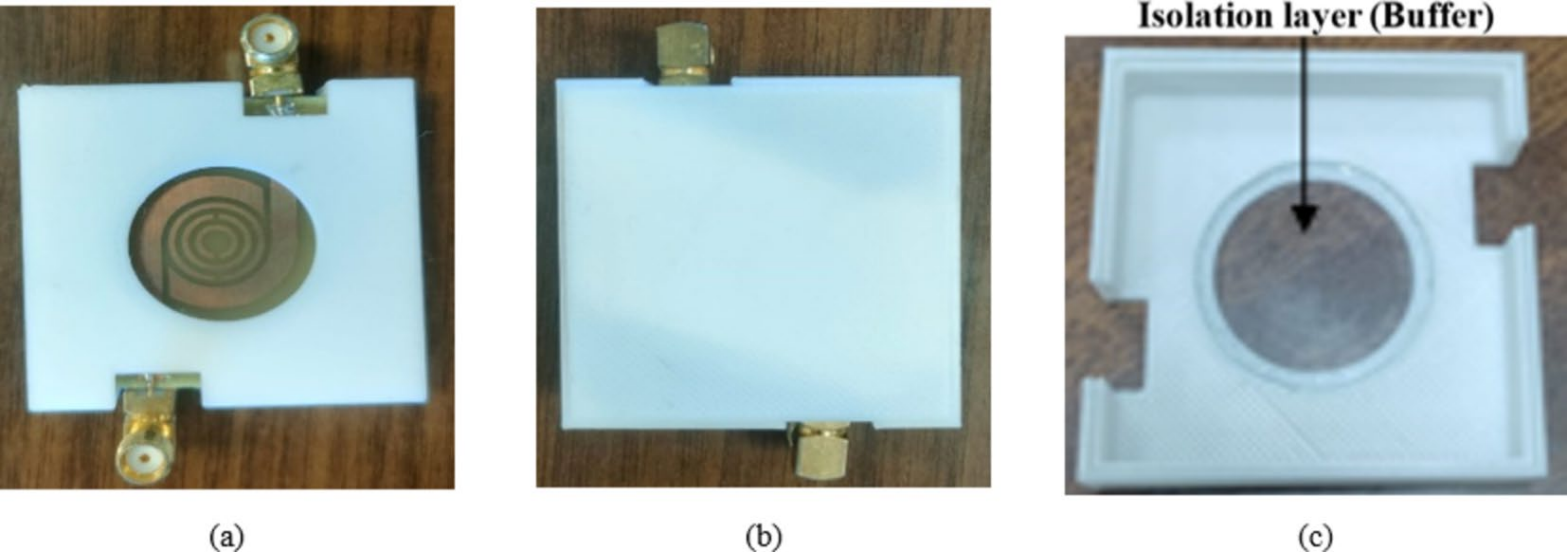
Housing of the proposed sensor, (**a**) Front view. (**b**) Back view and (**c**) Detailed view.

Moreover, a housing for the proposed sensor is designed to ensure measurement stability and minimize external influences. It includes a fixed-position guide that secures the fingertip consistently, maintaining uniform contact pressure across trials, as shown in (Fig. 15).

The housing is also equipped with an isolation layer to reduce the impact of sweat on the sensor’s performance. These design features help to mitigate the effects of movement, pressure variations, and environmental factors, thereby improving the accuracy and reliability of the glucose measurements. By changing the size of the finger, it was found that changes in transmission are minimal once the finger size covers most of the sensor footprint. Thus, any recorded changes will be a direct reflection to changes in glucose levels. Given the recorded results and fabricating a housing with a membrane for finger placement. Different membrane sizes could be assembled with the proposed sensor to accommodate variations in the size of a finger. Thus, the person intended to use the proposed sensor for measuring glucose levels to select a membrane that fits the finger size and covers all sensing areas. The membrane fabrication cost is less than 5$ and can be further reduced under mass production. Also, the given cost is minimal given that the proposed technique is diverting measuring glucose from invasive to non-invasive instantaneously and safely.

**Fig. 16:**
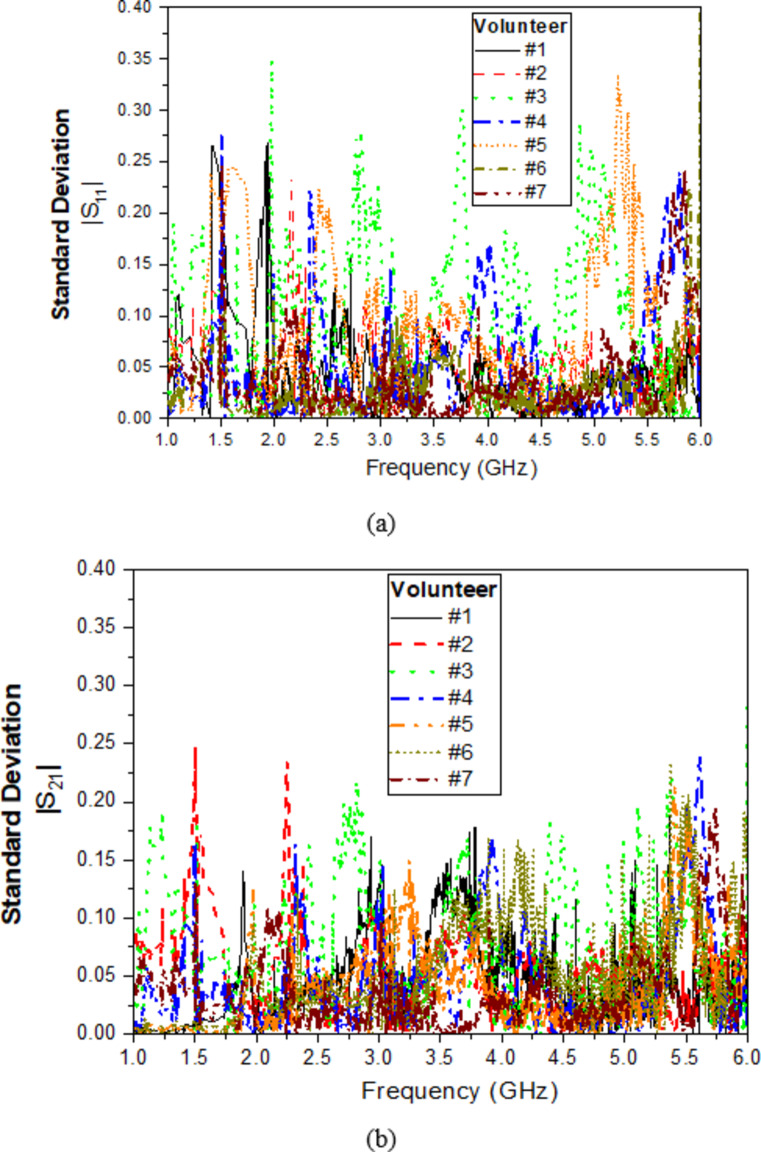
Standard deviation across 10 readings for each of 7 participants. (**a**) Reflection coefficient, (**b**) Transmission coefficient.

To confirm that the observed shifts in the S21 profile before and after fasting are directly related to changes in blood glucose levels (BGL), several measurements were implemented to minimize the influence of external factors. All measurements were conducted in a controlled environment, with participants remaining seated and instructed to avoid finger movement, thereby reducing potential impacts from motion and temperature fluctuations. Additionally, the sensor housing incorporated a fixed-position guide, as shown in (Fig. 15c), to ensure uniform fingertip finger placement and consistent contact pressure, minimizing variations due to fingertip positioning. Furthermore, 7 volunteers each conducted 10 measurements to obtain S11 and S21 readings, providing a thorough assessment of the sensor’s repeatability across various individuals. The details of the volunteers are described as shown in (Table 10). Figure 16 illustrates the standard deviation for each subject’s repeated readings of S11 and S21, both presented as magnitudes. The data shows that S21 exhibited a lower standard deviation than S11, indicating that the S21 readings have higher repeatability and are less susceptible to measurement variability. Thus showing greater robustness. This stability was further validated by correlating S21 measurements with conventional glucometer readings, which demonstrated a strong correlation with blood glucose levels (BGL). Consequently, the observed S21 shifts were primarily attributed to BGL changes rather than external factors, affirming the reliability of S21 for this application.

**Table 10:** Diversity of the volunteers involved in the experiment.

Volunteer #	1	2	3	4	5	6	7
Gender	Male	Female	Female	Female	Male	Female	Female
Age	38	46	50	26	29	25	30
With/wihtout diabetes	Without	Without	With	Pregnant & without	With	Without	Without

Incorporating machine learning into the realm of non-invasive blood glucose monitoring offers a powerful enhancement to the accuracy and reliability of diabetes detection and management^51^. By leveraging advanced algorithms, we can analyze the complex data obtained from the DBBPF sensor and improve the differentiation between diabetic and non-diabetic individuals. By exploring various machine-learning techniques, and evaluating their effectiveness in processing the sensor’s S-parameter signals and other relevant features^52^. Through a comparative analysis of algorithms such as support vector machines, decision trees, k-nearest neighbors, CatBoost Classifier, and neural networks we aim to identify the most efficient method for accurate glucose level classification and diabetes prediction, thereby advancing the potential of continuous non-invasive monitoring systems.

To evaluate the effectiveness of machine learning algorithms in detecting diabetes, we utilized a comprehensive dataset comprising 2003 records with six attributes directly related to diabetes risk. These attributes include the frequency of S11 magnitude, S11 phase, S21 magnitude, S21 phase, and the diabetes outcome. The diabetes outcome variable is binary, with a value of 0 indicating non-diabetes and 1 indicating diabetes as shown in (Table 11). The S-parameter data was extracted using a VNA or Nano VNA, providing critical inputs for the machine learning models. Table 11 provides a detailed description of this dataset. By analyzing these parameters, we can train various algorithms to accurately classify individuals as diabetic or non-diabetic, thus enhancing the utility of the dual-band bandpass filter sensor in practical diabetes management applications.

**Table 11:** The attributes of the diabetes dataset.

Attribute	Frequency	S11_magnitude	S11_phase	S21_magnitude	S21_phase	Diabetes outcome
Measure	GHz	dB	deg.	dB	deg.	(0 = non-diabetes)/(1 = diabetes)

The preprocessing steps are applied to the dataset to prepare it for machine learning model training. Preprocessing is crucial for ensuring the quality and effectiveness of the models, and addressing issues such as missing values, scaling, and data splitting^53^. A common challenge in data-driven fields is handling missing data, which can arise due to various reasons like limitations in data collection techniques or human error. Missing data refers to the absence of values for certain variables in the dataset. To address this, we examined each column with missing values to understand the reasons behind these gaps and replaced the missing values accordingly to maintain dataset integrity^54^. Normalization, a form of feature scaling, transforms the range of dataset features to a standard scale. This step is essential as our dataset includes features with varying ranges. By normalizing the data, we enhance model performance and accuracy, particularly because the data does not follow a Gaussian distribution. We employed min-max normalization, transforming the minimum value of each feature to 0, the maximum value to 1, and other values to a decimal between 0 and 1 as Eq. (5)^55^.

5 $${X_{norm}}=\frac{{X - {X_{\hbox{min} }}}}{{{X_{\hbox{max} }} - {X_{\hbox{min} }}}}$$

Data splitting involves dividing the dataset into multiple subsets, this involves a two-part split where one subset is used for training the model and the other for testing its performance. The training dataset is used to develop and refine the models, allowing for parameter estimation and performance comparison across different models. After training, the testing dataset evaluates the model to ensure it works correctly. In this study, we split the data with 70% allocated for training and 30% for testing. Data shuffling is a crucial preprocessing technique often used to enhance model learning. It addresses potential problems caused by patterns in the sequential order of training samples, which can lead to overfitting. By shuffling the data, we reduce bias during training, ensure randomness in batch selection, and prevent the model from learning any order-based patterns. This step is vital for creating a more robust and generalizable model.

**Fig. 17:**
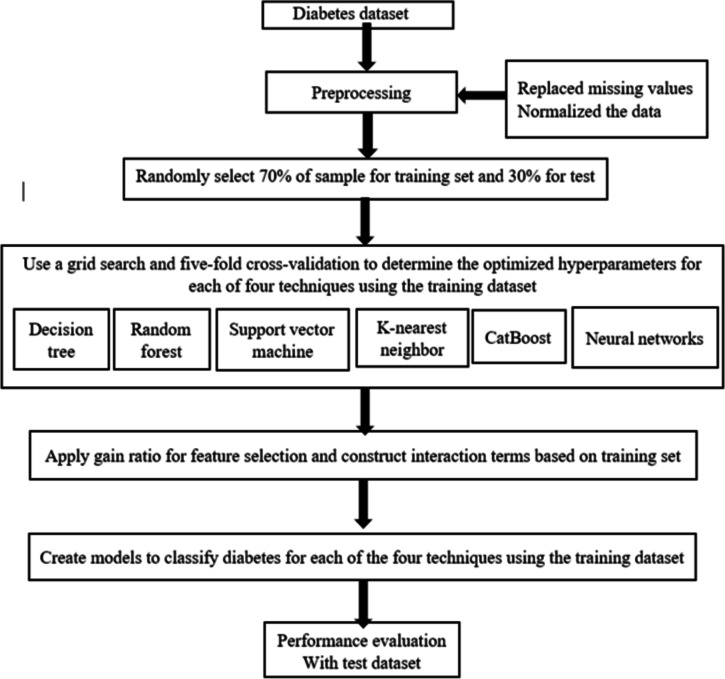
Flowchart of the utilized signal processing and machine learning models.

**Fig. 18:**
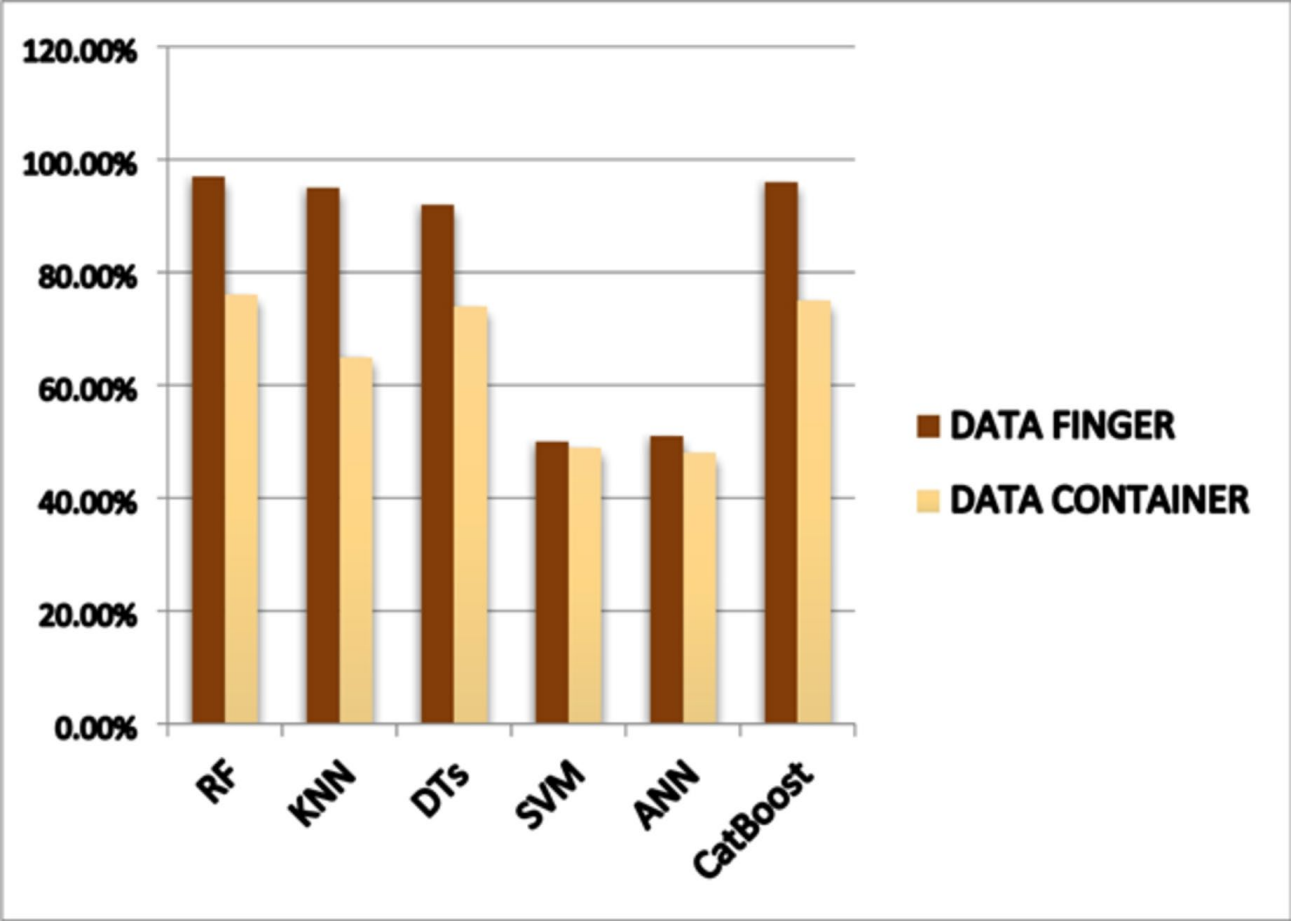
Performance comparison of machine learning models for diabetes prediction.

This flowchart in (Fig. 17) outlines the machine learning process for classifying diabetes using the described diabetes dataset. The process begins with preprocessing steps such as replacing missing values and normalizing the data. The dataset is then split into training (70%) and test (30%) sets as shown in (Fig. 18). The training data is used to optimize hyperparameters for six different machine learning techniques (decision tree, random forest, neural networks, K-nearest neighbor, CatBoost, and support vector machine) through grid search and cross-validation. Feature selection is performed using the gain ratio, and interaction terms are constructed. Subsequently, models are created for each technique using the training data, and their performance is evaluated with the test dataset.

By adopting machine learning algorithms, the results of this study models with interaction terms have better classification performance than those without interaction terms for all machine learning techniques. Among the proposed models with interaction terms, are random forest classifiers and CatBoost as shown in (Table 12). The measurement results of the finger are better than the container due to the type of finger-fat percentage - sweat. A comparative analysis of six machine learning models (RF, KNN, DTs, SVM, ANN, and CatBoost) based on their performance across two metrics: data finger and data container. Generally, the data finger metric (depicted in brown) shows higher performance compared to the data container (lighter yellow) across most models. Random Forest (RF) and CatBoost exhibit the strongest performance, achieving values close to or reaching 97% for data finger as shown in (Fig. 18), SVM and ANN demonstrate lower overall performance, hovering around 50% for both metrics. Decision Trees and KNN display moderate to high performance, particularly notable in the DATA FINGER metric. This chart enables a straightforward visual comparison of how effectively these models perform across the specified metrics, suggesting their suitability for a classification or prediction task. It’s critical to remember that these dyskinetic classifications are only broad recommendations and that individual circumstances, such as different glucose concentrations of people with diabetes, make sure their blood glucose levels are within their goal range, and to take the necessary steps to properly control their glucose levels, regular blood glucose monitoring is vital. The concentrations are compared with each other to avoid the problem of overlapping curves and to obtain the best possible result as shown in (Table 13).

**Table 12:** Classification for different machine learning techniques.

Technology	RF	KNN	DTs	SVM	ANN	CatBoost
First scenario (finger) %	97	95	92	50	51	96
Second scenario (container) %	76	65	74	49	48	75

**Table 13:** Classification for different machine learning techniques.

Technology	RF	KNN	DTs	SVM	ANN	CatBoost
First scenario (finger) %	80	75	85	60	66	88
Second scenario (container) %	**75**	70	80	50	60	78

Table 13 shows the percentages of success in differentiating between selected glucose concentrations are moderate around 50 to 88%, and CatBoost and RF algorithms achieved good percentages. Table 14 shows the percentages of success in differentiating between ionized water and selected glucose concentration. Table 14 indicates that as the range of concentration increased, the percentage increased.

**Table 14:** Classification comparison between ionized water and the other concentrations.

Technology	RF	KNN	DTs	SVM	ANN	CatBoost
0–50	58	50	50	46	45	60
0–100	62	66	67	47	50	69
0–150	80	72	68	60	65	82
0–200	87	75	79	75	76	85
0–350	90	80	82	79	86	95

Regression is a statistical method for simulating the connection between a dependent variable and one or more independent variables in regression analysis. Three types of algorithms are used: Support vector machine (SVM), CatBoost Classifier, which gives the best values of classification, and linear regression.

The regression is used to estimate the glucose concentration values based on observable data and to comprehend the kind and strength of the relationship between variables. It was applied A dual-band filter by selecting the minimum frequency with the minimum magnitude as shown previously in (Fig. 13). Then it is started interpolating all parameters between the concentrations to obtain a continuous representation for regression between six points at each minimum percentage as shown in (Table 15).

**Table 15:** S parameters of 6 points regression.

Parameters		SVM	CatBoost	Linear regression
S21	1.5 GHz	34	34	0.0005
	2.5 GHz	60	60	0.00074
S11	2.1 GHz	30	30	0.0002
	4.8 GHz	12	12	0.00001

## Conclusions

This paper presents a non-invasive microwave split ring resonator to sense glucose solutions at varying concentrations. dual band pass band filter based on split ring resonator system. Our paper offers a high Q-factor, low-profile microwave sensor simulation technique. Additionally, it provides a workable solution for the biomarker detection method for continuous monitoring of diabetes patients by using a non-invasive system. It is the capacity to detect minute variations in glucose concentrations surrounding the sensing region by using both simulation and measurement. The link between glucose concentration, S-parameter magnitude, phase, and resonant frequency were used to determine the biosensing response. With a sensitivity of 0.00067 degrees/(mg/dL) and 2.026 MHz/(mg/dL), the suggested device can detect all glucose levels linked to diabetes, including hypoglycemia, normoglycemia, and hyperglycemia. For instance, the proposed dual bandpass filter has a sensitivity of 0.011 dB/(mg/dL), which is higher than that of current microwave sensors. Its compact size, low cost, and straightforward design make it an excellent choice for a preliminary blood glucose test. Furthermore, a machine learning approach is introduced to enhance the effectiveness of the non-invasive microwave resonator for glucose sensing. By integrating machine learning techniques, such as Random Forest and CatBoost classifiers, with the proposed sensor system, it aims to improve the accuracy and reliability of glucose concentration detection in diabetic monitoring. The machine learning models leverage data from the sensor’s S-parameter measurements and glucose levels to provide robust predictions across varying glucose concentrations, supporting its application in continuous health monitoring systems.

## Electronic supplementary material

Below is the link to the electronic supplementary material.

Supplementary Material 1

## Data Availability

The data that support the findings of this study are available on request from the corresponding author Dalia Elsheakh. The data are not publicly available due to privacy or ethical restrictions.
